# Human motor cortex relies on sparse and action-specific activation during laughing, smiling and speech production

**DOI:** 10.1038/s42003-019-0360-3

**Published:** 2019-03-26

**Authors:** Markus Kern, Sina Bert, Olga Glanz, Andreas Schulze-Bonhage, Tonio Ball

**Affiliations:** 1 Medical AI Lab, Department of Neurosurgery, Medical Center – University of Freiburg, Freiburg, 79106 Germany; 2 Neurobiology and Biophysics, Faculty of Biology, University of Freiburg, Freiburg, 79104 Germany; 3 Epilepsy Center, Department of Neurosurgery, Medical Center – University of Freiburg, Freiburg, 79106 Germany; 4 BrainLinks-BrainTools Cluster of Excellence, University of Freiburg, Freiburg, 79110 Germany; 5 Hermann Paul School Linguistics, University of Freiburg, Freiburg, 79085 Germany; 6 GRK 1624, University of Freiburg, Freiburg, 79098 Germany

**Keywords:** Motor cortex, Brain-machine interface

## Abstract

Smiling, laughing, and overt speech production are fundamental to human everyday communication. However, little is known about how the human brain achieves the highly accurate and differentiated control of such orofacial movement during natural conditions. Here, we utilized the high spatiotemporal resolution of subdural recordings to elucidate how human motor cortex is functionally engaged during control of real-life orofacial motor behaviour. For each investigated movement class—lip licking, speech production, laughing and smiling—our findings reveal a characteristic brain activity pattern within the mouth motor cortex with both spatial segregation and overlap between classes. Our findings thus show that motor cortex relies on sparse and action-specific activation during real-life orofacial behaviour, apparently organized in distinct but overlapping subareas that control different types of natural orofacial movements.

## Abstract (web summary)

Markus Kern et al. use subdural recordings to understand how the human motor cortex is engaged during orofacial motor behavior such as speech production, laughing, and smiling. They find the motor cortex makes use of sparse and action-specific activation during these behaviors, which are organized in distinct but overlapping subareas.

## Introduction

Our ability to control orofacial movements with high precision is fundamental to verbal and non-verbal communication in everyday life. This includes voluntary motor control during speech production and deliberate laughing or smiling. Orofacial actions that are produced with less volitional control, such as the so-called emotion-driven Duchenne-type smiling or lip licking, also act as important communication signals in social and affective bonding^1,2^.

The first endeavours to functionally map the human cerebral cortex were undertaken with direct electrical stimulation mapping during awake neurosurgery^3,4^. Motor responses of the face and mouth including the tongue and lips were evoked from a strikingly large portion of the human primary/premotor motor cortex extending several centimetres along the central sulcus. Particularly the tongue and the lips were found to be spatially over-represented given their small actual size relative to other muscular effectors. This over-proportional representation has been commonly assumed to reflect the particularly fine-grained motor control of these oral muscular effectors in everyday behaviour, such as during speech production. However, many questions still remain on exactly how the motor control of natural orofacial movements is implemented in the human primary/premotor motor cortex.

While an activated mouth motor cortex was repeatedly reported in the form of gamma activity in electrocorticography (ECoG) during experimental^5–13^ as well as non-experimental^12,14^ overt speech production, recent findings raise doubts if the mouth motor cortex is necessarily involved in controlling natural smiling and laughing. According to refs. ^15–17^, emotionally driven smiling and laughing engages a different neural pathway than the kind of smile and laughing that are deliberately produced without a strong emotional drive. While the voluntary pathway comprises the premotor/primary motor cortex among several other brain areas, the emotional pathway does not comprise the motor cortex. Thus, in non-experimental conditions, smiling/laughing, at least if emotionally driven, could be performed without the need of an activated mouth motor cortex. The same could also apply to other orofacial actions that are produced with less volitional effort during natural behaviour, e.g., like lip-licking.

Assuming that the mouth motor cortex is involved in the control of such natural orofacial movements, also several questions remain. For example, is there a sub-regional specialization to major classes of natural motor behaviour like speech production vs. non-speech-related orofacial movements? And if so, on which spatial scale is such a potential action-based or task-specific organization principle implemented in human mouth motor cortex? Beyond electrical stimulation mapping to identify the mouth motor cortex, addressing these questions would require a functional mapping of brain activity with high spatial resolution during complex, real-life orofacial motor behaviours, which is methodologically challenging.

Complementary to experimental studies, recent studies show that such functional investigation of non-experimental, real-life behaviour can be successfully implemented leveraging intracranial recordings. Thus, natural movements have been investigated in the monkey brain^18,19^, revealing striking differences in the firing behaviour of motor cortical cells compared to those seen during constrained experimental conditions. In humans, however, the neural basis of motor control during real-life behaviour is largely unexplored^20^. Recent studies took advantage of ECoG recordings directly obtained from the brain surface to study the neural basis of real-life movements of the upper^14,21^ and lower extremities^14^, as well as of eye movements^22^ and non-experimental speech production^12,14,23–25^. But it remains unclear how major classes of orofacial actions that play a crucial role in our daily life and social interaction, including laughing and smiling, are controlled by the human mouth motor cortex in real-life conditions.

Better insights into how the human motor cortex controls natural orofacial actions could also lead to novel applications of brain−computer interfaces (BCI). The main focus of BMI/BCI research on motor control has so far been hand and arm movements, to decode these patterns by machine-learning techniques as a basis for neural motor prostheses, e.g., for patients with paralysis of the upper extremities^26^. Other studies have tried to reconstruct speech output from brain signals, opening new communication channels for locked-in patients^27^. Facial paralysis such as after stroke, head trauma, head tumour or infection/inflammation of the facial nerve, however, also affects a large number of individuals and can severely limit the freedom of facial expression and compromise the quality of social life of affected patients. It is currently not clear whether BCI concepts developed for restoration of upper-extremity movement can be applied to natural orofacial motor behaviours, and how and to what degree smiling vs. laughing vs. other orofacial movements is decodable from cortical brain signals.

In light of this, we explored cortical brain activity during the production of four distinct orofacial movement classes as they occurred during non-experimental, real-life conditions: lip licking, speech production, laughing, and smiling. To probe the cortical activation during these conditions, we used ECoG that offers high spatial resolution comparable to hemodynamic methods within the region of the cortical surface covered by electrode contacts^28^. ECoG is also much more robust against artefacts of extracranial origin compared to non-invasive EEG^29^. Thus, ECoG is optimally suited to study the neural basis of natural behaviour in general, and orofacial movement in particular. ECoG gamma band activity was previously found to be a useful marker of cortical brain activity during non-experimental, real-life conditions^14,25^; thus, we focused our ECoG analyses on the event-related spectral modulations in the gamma frequency range^30–34^ in addition to event-related potentials (ERPs)^35–37^.

We show that real-life orofacial movements, including natural smiling/laughing as well as lip-licking can indeed be accompanied by strong gamma band modulations within the mouth motor cortex. For each orofacial movement class only a class-specific subset of electrode contacts within the mouth motor cortex recorded gamma band modulations, pointing to a sparse and action-specific functional organization of the human mouth motor cortex. Finally, we showed that the intracranial-recorded brain activity in mouth motor cortex of orofacial movements belonging to different movement classes contained sufficient information to be correctly classified on a single-trial basis.

## Results

### Electrocortical stimulation mapping (ESM)

Four different types of mouth-related motor responses were elicited during ESM in the six participants (P1−P6), namely motor responses of the tongue, the lips, the palate and the jaw. Mouth motor responses mostly occurred upon stimulation of the cortex anterior to and directly on the central sulcus, while mouth sensory responses were observed solely posterior to the central sulcus. Overall, mouth-related responses during ESM were mostly motoric and of the tongue or the lips, with the exception of two palate- and one jaw-related electrode contact.

**Fig. 1:**
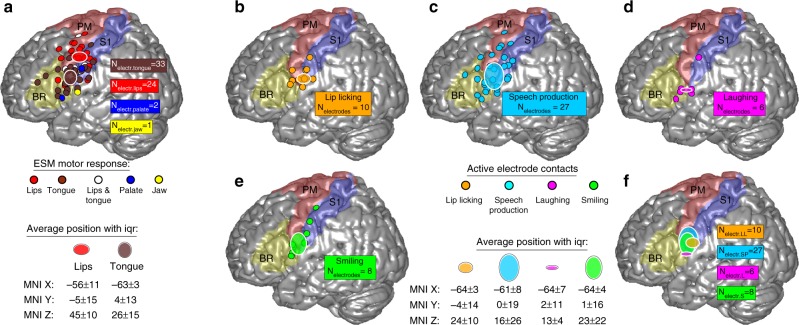
Electrode contacts positions with (**a**) mouth motor function and with (**b**–**f**) event-related gamma power modulation. **a** Circles indicate the individual electrode contacts positions with mouth motor function (according to the results of the ESM) of all six participants, superimposed on a standard brain. The respective mouth-related body parts are colour-coded as following: the lips (red), the tongue (brown), the palate (blue) and the jaw (yellow). The number of electrode contacts with the respective ESM motor response is listed as *N*_electr_motor_function_. Ellipsoids indicate average positions of electrode contacts with lips (red-white) or tongue (brown-white) motor function; with the centre and size of the ellipsoids indicating the median and interquartile range of coordinates from the respective electrode contacts in MNI space. Exact values are listed beneath the figure, the respective MNI *y*- and *z*-coordinates are shown graphically. The typical extent of premotor cortex (PM; red area), primary somatosensory cortex (S1; blue area) and Broca’s area (BR; yellow area) are shown based on the anatomical probability information of the SPM Anatomy Toolbox. **b**–**e** Overview of significant (sign test; *p* < 0.01) event-related gamma power modulation within the ESM-defined mouth motor cortex, superimposed on a standard brain surface with the number of active electrode contacts listed as *N*_electr_event_. Small coloured dots indicate active electrode contacts, i.e., contacts that recorded significant movement-related gamma power modulations in the respective movement class. Large ellipsoids are centred at the average position (median over electrode contacts) and are scaled proportional to the interquartile range as a measure of spatial variance of the MNI *y*- and *z*-coordinates. Exact MNI coordinates are listed between (**e**) and **f**. Ellipsoids are overlaid for all movement classes in the bottom right panel (**f**) for a better overview. ESM electrocortical stimulation mapping

On the group level, the average position of electrode contacts with lips motor function (red-white striped oval; Fig. 1a) was located significantly (sign test; *p* < 0.01) more dorsal (*p* = 0.00007) and more medial (*p* = 0.0005) than the average position of electrode contacts with tongue motor function (brown-white striped oval; Fig. 1a). The Euclidean distance between the average position of electrode contacts with lips motor function and of tongue motor function (MNI coordinates are listed beneath Fig. 1a) was approx. 22 mm. Electrode contacts with palate or jaw motor function were too few to allow statistical evaluation.

As post-implantation MRIs were available in all participants included in the present study, it was also possible to combine precise individual morphological information derived from these imaging data with probabilistic anatomical information. Hence, we were able to delineate the regional assignment of individual electrode contacts. This regional assignment revealed that lips- and tongue motor responses were not restricted to premotor cortex (red area; Fig. 1a) and primary sensory cortex (blue area; Fig. 1a), but also occurred in Broca’s area, i.e., in Brodmann’s area 44 and 45 (yellow area; Fig. 1a).

### Spatial distribution of event-related gamma modulations

Our analysis of event-related cortical activity during real-life orofacial movements revealed significant (sign test; *p* < 0.01) brain activity in all participants investigated. Active electrode contacts, i.e., electrode contacts at which significant event-related gamma band modulation was recorded, were mainly located within the ESM-defined mouth motor cortex. Within mouth motor cortex, overall ten electrode contacts were active during lip licking (Fig. 1b), 27 during speech production (Fig. 1c), six during laughing (Fig. 1d) and eight during smiling (Fig. 1e). The averaged MNI coordinates and the respective interquartile range of active electrode contacts located within the ESM-defined mouth motor cortex for all four movement classes are shown in Fig. 1f.

We observed no significant (*p* < 0.01) differences in any spatial direction (*x*, *y*, or *z*) when we compared the average position in MNI space of active, movement class-specific electrode contacts (two-sided Wilcoxon rank sum test) for each possible pair of orofacial movement classes. Only in the *z*-direction there was a tendency for laughing-related gamma band modulations to be different from the other orofacial movement classes. Exact *p*-values were: *p* = 0.025 (laughing vs. lip licking); *p* = 0.047 (laughing vs. speech production) and *p* = 0.075 (laughing vs. smiling).

Summarized, electrode contacts that recorded event-related gamma band modulation (active electrode contacts) were mainly located within the ESM-defined mouth motor cortex in all participants investigated. Although the respective subsets of active electrode contacts were not identical between movement classes, these subsets were located in the same overall cortical region without significant differences in any spatial direction (*x*, *y*, or *z*) when comparing the averaged position of the respective electrode contacts.

### Sparse cortical activity in mouth motor cortex

**Fig. 2:**
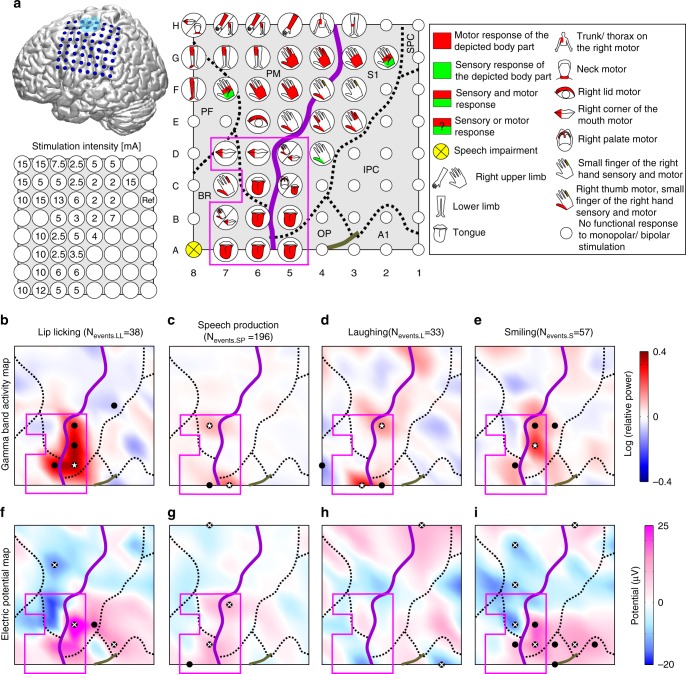
Electrocortical stimulation map and orofacial event-related brain activity maps of P1. **a** Position of the 8 × 8 electrode array visualized on a standard brain surface. Blue: seizure onset zone/area of multiple subpial transections. Electrocortical stimulation mapping (ESM): stylized body parts indicate ESM responses; motor and sensory responses shown in red and green, respectively. Magenta outline: borders of the ESM-defined mouth motor cortex. Purple and grey lines: central and lateral sulcus derived from individual post-implantation MRI, respectively. Black dotted lines: borders between cortical areas, determined by using the probabilistic anatomical maps from the Anatomy Toolbox 1.6 in SPM8. PF prefrontal cortex, BR Broca’s area, PM premotor cortex, S1 primary somatosensory cortex, IPC inferior parietal cortex, SPC superior parietal cortex. Stimulation intensity for all electrode contacts with ESM responses is shown beneath the standard brain. **b**–**e** Gamma band (55–200 Hz) activity maps related to the different orofacial movement classes; interpolated maps are shown (from left to right): lip licking (**b**), speech production (**c**), laughing (**d**) and smiling (**e**). Black dots indicate significant brain activity (sign test; *p* < 0.05 for <100 events and *p* < 0.0001 for >100 events; corrected for multiple comparisons), white stars indicate local maxima. **f**–**i** Electrical potential map related to the different orofacial movement classes; conventions as in **b**–**e**, but for the movement-related potentials (MRP)

Also on the single-participant level (shown exemplary for P1; Fig. 2), as expected the ESM-defined mouth motor cortex (magenta outline; Fig. 2a) was the main region of event-related cortical activity. Gamma band increases were observed within this region during all four orofacial movement classes, lip licking (Fig. 2b), speech production (Fig. 2c), laughing (Fig. 2d), and smiling (Fig. 2e). Crucially, there was no example in which two movement classes activated exactly the same set of electrode contacts; thus, in each case there was a unique spatial activity pattern within the mouth motor cortex associated with all of the orofacial movement classes studied here (Fig. 2b–e). Adjacent electrode contacts even with identical ESM motor responses were often active in different classes of natural orofacial motor behaviour.

Averaged over participants and orofacial movement classes, only 18% (std: 5%) of electrode contacts located within the mouth motor cortex were active. Thus, each orofacial movement class only sparsely activated the mouth motor cortex in the spatial domain. One concern here is that corrected tests could be too conservative, and uncorrected *p* values should be used to determine significance, to avoid false negatives and as the number of electrode contacts and hence of statistical tests was already reduced by restricting the analysis to mouth motor cortex. When using uncorrected *p* values the mouth motor cortex was still only sparsely activated, i.e., averaged over participants and movement classes only 25% (std: 5%) of the electrode contacts within the ESM-defined mouth motor cortex were active.

Another concern is that sparse activation was due to relatively low numbers of trials that could be obtained for certain movement classes: Speech production was the orofacial movement class with the highest number of active electrode contacts, on average 57% (uncorrected) of the electrode contacts located within the mouth motor cortex were active during this class. Speech production was also the orofacial movement class with the highest number of trials, on average 146 trials per participant, while the other movement classes had only 33 trials on average. A positive correlation (Spearman’s rank correlation coefficient; corrected: rho = 0.56, *p* = 0.0041; uncorrected: rho = 0.79, *p* = 0.00005) was observed between the number of trials of each movement class and the respective percentage of active electrode contacts located within mouth motor cortex. This correlation was mainly driven by the high number of trials and active electrode contacts in the speech production class. Thus, no significant correlation was observed between the number of trials and the percentage of active electrode contacts when using only the non-speech movement classes (uncorrected: rho = 0.37, *p* = 0.13).

When reducing the number of events in the speech production class to the average number of the remaining classes (33 trials) as well as using the same statistical threshold (*p* < 0.01, uncorrected) decreased the percentage of active electrode contacts during speech production from 57 to 32%. Nevertheless, speech production remained the orofacial movement class with the highest percentage of active electrode contacts within the EMS-defined mouth motor cortex, followed by laughing (14%), lip licking (16%) and smiling (13%). By using the reduced number of trials (33 trials) for the speech production class and the respective percentage of active electrode contacts, the correlation between the class-specific number of events and the respective percentage of active electrode contacts also diminished (rho = 0.25, *p* = 0.24).

In addition to the spectral power changes in the gamma band, we also evaluated brain activity reflected in the time-domain by movement-related potentials (MRPs, Fig. 2f–i). Comparable to the gamma band modulations, each orofacial movement class was accompanied by a unique MRP pattern with local maxima and minima at different electrode contacts. MRP phase reversal was typically close to the central sulcus (e.g., Fig. 2f, i).

Summarized, in our data the mouth motor cortex was only sparsely activated in the spatial domain. Speech production was the orofacial movement class that activates the largest subset of electrode contacts within the mouth motor cortex, even if the number of trials in this class is reduced to the average trial number of all other movement classes investigated here. Speech production was also the orofacial movement class that occurred most frequently compared to the non-speech classes in our data sets of real-life human behaviour, which is probably also the case under most circumstances in natural social behaviour.

### Event-related gamma band modulations at oral motor effectors

**Fig. 3:**
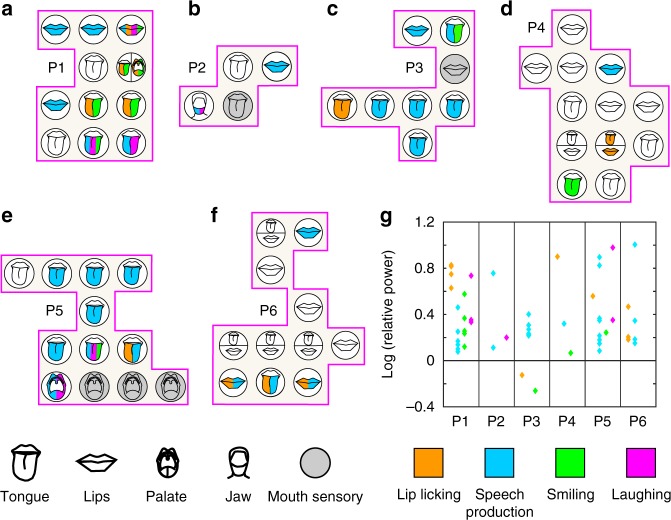
Event-related gamma band modulations and their corresponding ESM-defined oral motor effector. **a**–**f** For each participant, P1 (**a**)−P6 (**f**), all electrode contacts within the ESM-defined mouth sensorimotor cortex (pink border) are illustrated as circles with the respective ESM-defined mouth-related body part schematically indicated. Coloured symbols indicate significant (sign test; *p* < 0.01) orofacial movement-related gamma band activity at ESM-defined mouth motor-related electrode contacts: lip-licking = orange, laughing = pink, speech production = blue, smiling = green. Electrode contacts with sensory responses during ESM are shown in grey. **g** For each participant the strength of the event-related gamma power modulation for each active electrode contacts is shown. ESM electrocortical stimulation mapping

Up to this point, we addressed the mouth motor cortex as one homogenous area and observed movement-class-specific brain activity patterns within this area. As a next step, we analysed the exact ESM motor response of single active electrode contacts located within the mouth motor cortex in more detail (Fig. 3). We were interested whether electrode types, defined by their specific ESM-defined oral motor effector, were only activated by specific movement classes or rather by multiple or all studied movement classes.

As described above, the number of different electrode types, as defined by their respective ESM mouth-related motor response, varied strongly across participants with most electrode contacts showing tongue or lip ESM responses, with the exception of one palate motor-related electrode contact in P1 (Fig. 3a), one in P5 (Fig. 3e), and one jaw motor-related electrode contact in P2 (Fig. 3b). Thus not all mouth motor-related electrode types were available in every participant. Nevertheless, e.g., in P1 (Fig. 3a), every orofacial movement class activated at least one tongue motor- and at least one lips motor-related electrode contact. Although no other participant had both active electrode contacts with tongue and lips motor function at each single orofacial movement class, across participants all orofacial movements activated several ESM-defined tongue motor- and at least one lips motor-related electrode contact (see Table 1). These results reflect that both the tongue and the lips are involved in the execution of all studied orofacial movement classes.

**Table 1:** Proportion of electrode contacts with significant (sign test; *p* < 0.01) gamma band activity per electrode type and orofacial movement class on the group level

	Tongue electrodes	Lips electrodes	Palate electrodes	Jaw electrodes
Lip-licking	7/33	4/24	1/2	0/1
Speech production	15/33	10/24	1/2	1/1
Laughing	3/33	1/24	1/2	1/1
Smiling	6/33	1/24	1/2	0/1

With regard to palate motor-related electrode contacts, significant (sign test; *p* < 0.01) gamma activity was exclusively observed during lip licking and smiling in P1 (Fig. 3a), but not during speech production and laughing. In contrast, the single palate motor-related electrode contact in P5 (Fig. 3e) was exclusively activated during speech production and laughing. Thus, across participants, every orofacial movement class activated exactly one palate-related electrode contact, which, across participants, points to the involvement of the palate in every movement class studied.

The amplitude of the event-related gamma band modulations varied strongly over participants, orofacial movement classes and electrode contacts (Fig. 3g). The strongest event-related gamma power modulations were either observed during lip licking (P1 and P4), during speech production (P2, P3 and P6), or during laughing (P5). In P3 lip licking and smiling were accompanied by a spectral power decrease in the gamma band. The MNI coordinates, *z* scores, *p* values and the ESM results for each individual mouth motor electrode contact that was active during at least one type of orofacial movement are listed in Table 2.

**Table 2:** MNI coordinates of electrode contacts with significant (sign test; *p* < 0.01) gamma band effects within ESM-defined mouth motor cortex

	Electrode name	MNI coordinate (mm)			Area	*Z*-score (*p* value) Significant (sign test; *p* < 0.01) values shown in bold				ESM (intensity)
		*X*	*Y*	*Z*		Lip-licking	Speech	Laughing	Smiling	
P1	A5	−66	−3	14	OP	1.1 (2.6e^−01^)	**8.6 (5.5e** ^**−18**^ **)**	**5.2 (7.9e** ^**−09**^ **)**	−2.4 (1.6e^−02^)	Tongue (5 mA)
	A6	−64	7	14	BR	0.2 (8.7e^−01^)	**7.1 (1.5e** ^**−12**^ **)**	**5.2 (7.9e** ^**−09**^ **)**	**2.9 (3.2e** ^**−03**^ **)**	Tongue (5 mA)
	B5	−65	−5	24	S1	**6.0 (7.3e** ^**−12**^ **)**	0.5 (6.2e^−01^)	0.7 (4.9e^−01^)	**2.6 (7.5e** ^**−03**^ **)**	Tongue (6 mA)
	B6	−63	7	22	BR	**5.7 (2.8e** ^**−10**^ **)**	2.5 (1.2e^−02^)	−3.4 (3.2e^−04^)	**4.8 (7.5e** ^**−07**^ **)**	Tongue (6 mA)
	B7	−61	16	23	BR	0.2 (8.7e^−01^)	**3.4 (7.9e** ^**−04**^ **)**	0.7 (4.9e^−01^)	1.6 (1.1e^−01^)	Lips (10 mA)
	C5	−64	−7	33	S1	**6.0 (7.3e** ^**−12**^ **)**	0.0 (1.0)	1.0 (3.0e^−01^)	**4.8 (7.5e** ^**−07**^ **)**	Tongue + palate (3.5 mA)
	D5	−59	−10	44	S1	**6.0 (7.3e** ^**−12**^ **)**	**3.6 (2.7e** ^**−04**^ **)**	**3.8 (6.6e** ^**−05**^ **)**	**5.0 (1.5e** ^**−07**^ **)**	Lips (5 mA)
	D6	−56	2	46	PM	−0.8 (4.2e^−01^)	**7.6 (2.1e** ^**−14**^ **)**	2.4 (1.4e^−02^)	−1.9 (6.3e^−02^)	Lips (2.5 mA)
	D7	−52	13	43	BR	−1.8 (7.3e^−02^)	**2.8 (5.3e** ^**−03**^ **)**	−0.7 (4.9e^−01^)	−0.3 (7.9e^−01^)	Lips (10 mA)
**P2**	C7	−66	−3	10	CS	0.0 (1.0)	**11.4 (3.1e** ^**−30**^ **)**	**3.4 (2.7e** ^**−04**^ **)**	−0.5 (6.3e^−01^)	Jaw (4.5 mA)
	E8	−63	−14	37	S1	0.8 (4.5e^−01^)	**4.4 (1.3e** ^**−05**^ **)**	−0.5 (6.3e^−01^)	1.5 (1.4e^−01^)	Lips (12 mA)
**P3**	A4	−66	−14	26	OP	−0.5 (6.3e^−01^)	**6.2 (4.6e** ^**−10**^ **)**	1.4 (1.5e^−01^)	0.8 (4.4e^−01^)	Tongue (3 mA)
	B3	−61	−18	41	S1	1.0 (3.3e^−01^)	**5.1 (2.8e** ^**−7**^ **)**	2.0 (3.9e^−02^)	−1.1 (2.8e^−01^)	Tongue (15 mA)
	B4	−63	−10	36	S1	1.0 (3.3e^−01^)	**7.3 (2.2e** ^**−13**^ **)**	2.0 (3.9e^−02^)	1.4 (1.6e^−01^)	Tongue (8 mA)
	B5	−62	0	33	PM	1.9 (4.9e^−02^)	**6.8 (1.2e** ^**−11**^ **)**	2.0 (3.9e^−02^)	0.8 (4.4e^−01^)	Tongue (5 mA)
	B6	−62	7	32	PM	**−2.9 (2.3e** ^**−03**^ **)**	2.2 (2.8e^−02^)	0.3 (7.7e^−01^)	0.8 (4.4e^−01^)	Tongue (5 mA)
	D3	−50	−16	56	PM	0.0 (1.0)	**7.5 (5.6e** ^**−14**^ **)**	0.9 (3.9e^−01^)	**−2.9 (2.9e** ^**−03**^ **)**	Tongue (2 mA)
	D4	−49	−7	54	PM	−1.9 (4.9e^−02^)	**7.2 (8.7e** ^**−13**^ **)**	−2.6 (6.3e^−03^)	1.4 (1.6e^−01^)	Lips (10 mA)
**P4**	B7	−61	6	20	BR	−0.5 (6.4e^−01^)	0.0 (1.0)	0.0 (1.0)	**2.6 (8.6e** ^**−03**^ **)**	Tongue (13 mA)
	C6	−63	−6	32	S1	**4.0 (4.2e** ^**−05**^ **)**	2.1 (3.3e^−02^)	0.0 (1.0)	−0.8 (4.4e^−01^)	Tongue + lips (3 mA)
	E6	−52	−5	52	PM	−1.1 (2.7e^−01^)	**3.4 (6.1e** ^**−04**^ **)**	−1.7 (9.2e^−02^)	−1.6 (1.2e^−01^)	Lips (1.5 mA)
**P5**	E4	−54	28	26	BR	1.9 (5.7e^−02^)	**7.9 (2.2e** ^**−15**^ **)**	−0.5 (5.8e^−01^)	0.5 (6.0e^−01^)	Tongue (8 mA)
	E5	−57	16	33	BR	0.3 (7.9e^−01^)	**11.2 (5.4e** ^**−29**^ **)**	−1.3 (2.0e^−01^)	0.3 (7.9e^−01^)	Tongue (2 mA)
	E6	−61	4	36	PM	0.0 (1.0)	**16.6 (9.2e** ^**−62**^ **)**	0.2 (8.6e^−01^)	−0.5 (6.0e^−01^)	Tongue (2.5 mA)
	F5	−63	9	20	CS	2.4 (1.3e^−02^)	**5.2 (2.4e** ^**−07**^ **)**	−0.9 (3.6e^−01^)	−1.8 (6.7e^−02^)	Tongue (3.5 mA)
	G4	−60	16	7	BR	0.3 (8.0e^−01^)	**2.9 (3.9e** ^**−03**^ **)**	0.5 (5.8e^−01^)	0.0 (1.0)	Tongue (4 mA)
	G5	−64	8	11	CS	2.4 (1.3e^−02^)	**16.3 (4.9e** ^**−60**^ **)**	**3.5 (3.2e** ^**−04**^ **)**	**3.1 (1.5e** ^**−03**^ **)**	Tongue (2.5 mA)
	G6	−65	−3	16	OP	**2.9 (1.8e** ^**−03**^ **)**	**13.9 (3.6e** ^**−44**^ **)**	−0.9 (3.6e^−01^)	0.8 (4.4e^−01^)	Tongue (5 mA)
	H4	−58	12	3	LS	0.0 (1.0)	**3.8 (1.2e** ^**−04**^ **)**	**3.5 (3.2e** ^**−04**^ **)**	0.5 (6.0e^−01^)	Palate (15 mA)
**P6**	B4	−66	−13	24	OP	**2.6 (8.6e** ^**−03**^ **)**	**3.5 (3.9e** ^**−04**^ **)**	−1.8 (6.3e^−02^)	−1.4 (1.5e^−01^)	Lips (9 mA)
	B5	−64	1	23	CS	**5.7 (1.4e** ^**−09**^ **)**	**2.9 (3.7e** ^**−03**^ **)**	0.0 (1.0)	1.4 (1.5e^−01^)	Tongue (3 mA)
	B6	−62	9	18	BR	**3.4 (5.8e** ^**−04**^ **)**	**4.7 (9.4e** ^**−07**^ **)**	1.8 (6.3e^−02^)	−1.4 (1.5e^−01^)	Lips (3 mA)
	F4	−39	−10	65	PM	−0.5 (6.0e^−01^)	**6.5 (1.1e** ^**−13**^ **)**	0.0 (1.0)	−1.4 (1.5e^−01^)	Lips (1.5 mA)

*BR* Broca’s area, *CS* Central Sulcus, *IPC* inferior parietal cortex, *LS* lateral sulcus, *OP* parietal operculum, *PM* premotor cortex, *S1* primary somatosensory cortex, *ESM* electrocortical stimulation mapping

Summarized, across participants we observed movement-related gamma band modulations, mostly increases in gamma power, at all four types of ESM-defined mouth motor-related electrode contacts. Across participants each movement class studied here activated every type of mouth motor-related electrode contact (with the only exception of the single-electrode contact with jaw motor function found in P2, which was activated during speech production and laughing only).

### Spatial overlap/segregation of movement class-related gamma

With regard to the spatial distribution of gamma modulations on the group level, on the one hand, we observed clear differences between the orofacial movements classes studied, on the other hand, brain activity also overlapped in a considerable number of electrode contacts.

**Fig. 4:**
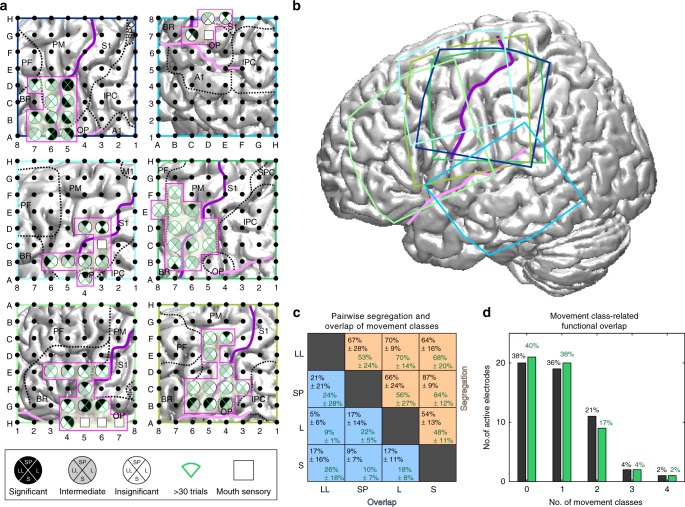
Spatial overlap/segregation of movement class-related gamma band activity patterns. **a** Spatial overlap/segregation (see Methods) of orofacial movement-related gamma band activity patterns within mouth motor cortex on a single-participant level; P1−P6 from top left to bottom right. Circles represent electrode contacts with mouth motor function within the borders of the ESM-defined mouth sensorimotor cortex (solid magenta outline). Each segment of a circle represents a specific orofacial movement class; leftward segment: lip licking, upper segment: speech production, rightward segment: laughing and lower segment: smiling. The greyscale of the segments indicates the significance with respect to movement-related gamma band activity at the respective electrode contact. Segments corresponding to movement classes with more than 30 trials from a participant are marked by green frames. **b** Position of the 8 × 8 electrode arrays of all six participants visualized on a standard brain surface. Orofacial movement classes are abbreviated as following: lip-licking (LL), speech production (SP), laughing (L) and smiling (S). Solid purple and pink lines indicate the course of the central and lateral sulcus, respectively. **c** Average pairwise segregation and overlap values and their standard deviation for all possible pairs of orofacial movement classes. In black: mean over participants and all orofacial movement classes; in dark green: mean over participants only for orofacial movement classes with >30 trials. **d** Movement-class-related functional overlap is shown as number of electrodes (*y*-axis) where significant (sign test; *p* < 0.01) gamma band activity was recorded for 0–4 movement classes (*x*-axis). Overall, the spatial overlap of movement class-related gamma band activity was rather low. In black: all movement classes; in green: only for movement classes with >30 trials

In a next step, we investigated the spatial overlap/segregation of movement class-related gamma band modulations on the single-electrode level based on all mouth motor-related electrode contacts of the six participants investigated (Fig. 4). For each electrode contact within the mouth motor cortex (Fig. 4a; pink boxes) of the respective electrode grid (location of the respective grids in Fig. 4b), the movement-related gamma band modulation of each movement-class was classified based on their statistical significance (see methods) into (I) significant (*p* < 0.01; uncorrected), (II) intermediate (not significant, but *p* < 0.3), and (III) insignificant (*p* > 0.3).

Based on this classification, we calculated for each possible pair of movement classes its *pairwise overlap value* as the percentage of electrode contacts within the mouth motor cortex that recorded converging (both significant or both intermediate) gamma band modulations across two movement classes, as well as the *pairwise segregation value* (different statistical classification) as the percentage of electrode contacts that recorded diverging gamma band activity across two movement classes. The resulting *pairwise segregation values* were considerably larger than the respective *pairwise overlap values* for every possible pair of movement classes (Fig. 4c, black numbers). This also holds true when only orofacial movement classes that were performed more than 30 times by a participant (marked with green frame in Fig. 4a) were taken for the analysis (Fig. 4c, dark green numbers). The highest *pairwise segregation value* was observed between speech production and smiling, the lowest one between laughing and smiling.

Overall, 20 electrode contacts out of the total of 53 mouth motor-related electrode contacts were inactive, i.e., did not record significant (*p* < 0.01; uncorrected) gamma band activity during any orofacial movement class (Fig. 4d). For analysis with always >30 trials, results were very similar (21 electrodes); this in the following paragraph we report the findings for analysing the full data set. There, 19 electrode contacts were active during one movement class only, while 11 electrode contacts were active during two movement classes, two contacts during three classes and one electrode contact during all four classes. This shows an overall rather low functional overlap of only 26%, while 38% of the covered mouth motor cortex was not active at all during any orofacial movement. Excluding this inactive mouth motor cortex results in a functional overlap within the active mouth motor cortex of 58% and a corresponding segregation of 42%.

### Single-trial decoding of natural orofacial movements

Our observation of unique movement-class-specific spatial patterns of brain activity in the trial-averaged data led us to ask whether the four different orofacial movement classes can be decoded on a single-trial basis. If so, this would encourage further investigations towards the possibility of restoring orofacial movements using brain interfaces.

We performed three versions of the single-trial decoding analysis: first, based on brain signals captured by individual electrode contacts. This analysis enables the spatial allocation of the sources of decodable information, but cannot exploit synergies between the signals from multiple electrode contacts. Therefore, we also carried out a multivariate decoding on the cortical motor signals of all electrode contacts within the mouth motor cortex. Finally, we tested the effect of averaging over subareas within mouth motor cortex, which may be useful for improving decoding accuracies especially if the signals are correlated but the noise is not and thus cancels out through the averaging. All variants of the decoding procedure were compared for gamma band activity and time-domain potential features alone, as well as the combination of both, using a regularized linear discriminant classifier (rLDA, see Methods).

**Fig. 5:**
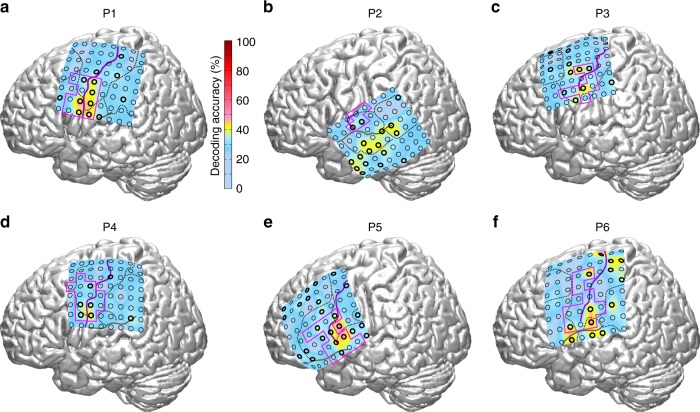
Maps of decoding accuracy for discriminating the four movement classes by gamma band activity. **a**–**f** Position of the 8 × 8 electrode arrays of all six participants visualized with the interpolated colour-encoded decoding accuracy obtained from the individual electrode contacts (circles), superimposed on a standard brain surface. Electrode contacts with significant (corrected for multiple comparisons; *q* < 0.05) decoding accuracy are marked by circles with thicker black outlines. Dashed lines: borders between cortical areas; solid purple and brown line: course of the central sulcus and lateral sulcus, respectively; solid magenta outline: borders of the ESM-identified mouth sensorimotor cortex. Decoding accuracy within the mouth sensorimotor cortex reached significance in all six participants. ESM electrocortical stimulation mapping

**Fig. 6:**
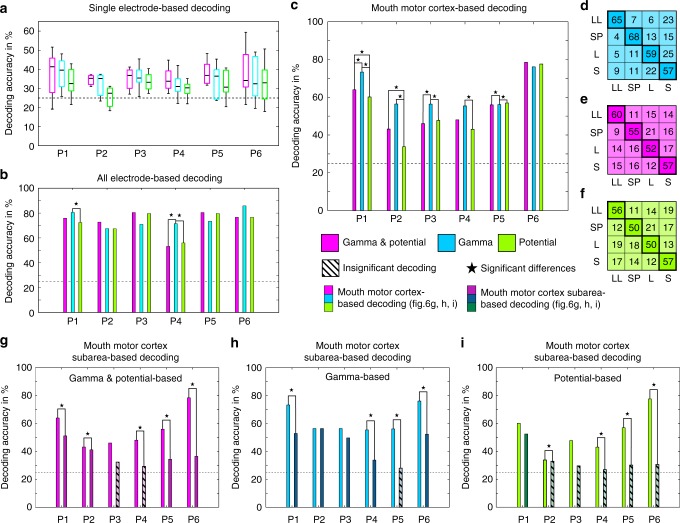
Decoding accuracies based on different signal features. **a** Decoding accuracy of single-electrode-based decoding using gamma band activity (cyan), potentials (green), or the combination of both (purple) for all participants. The central mark is the median, the edges of the box are the 25th and 75th percentiles, the whiskers extend to the most extreme datapoints. The dashed line indicates chance level (25%). **b** Decoding accuracy from all grid electrode contacts together. Decoding accuracy was significantly above chance (*p* < 0.01, permutation test) in all cases. Significant (*p* < 0.01, sign test) differences in decoding accuracy obtained with different signal components within participants are marked with a star. **c** Decoding accuracy from all electrode contacts in mouth motor cortex together. Decoding accuracy was significantly above chance (*p* < 0.01, permutation test) in all cases. Gamma band-based decoding significantly outperformed the alternatives in four out of six participants. **d** Confusion matrix of the classification based on gamma band activity and potential, **e** gamma band activity only and **f** potential only, as the mean over participants. For each orofacial movement class (lip-licking (LL), speech production (SP), smiling (S) and laughing (L)), the respective row indicates the percentage of classifications for the movement class in the respective column. Thus, values along the highlighted diagonal indicate the percentage of correct classifications for each movement class, and all off-diagonal values are classification errors. As illustrated by the confusion matrices, gamma band activities yielded the overall best performance due to higher accuracies for lip licking, speech production and laughing, while the decoding accuracy for smiling was the same in all cases. **g−i** Decoding accuracy based on either all mouth motor-related electrode contacts together or using the mean over each mouth motor subarea, i.e., the activity was averaged over electrode contacts with identical oral effector. Decoding accuracy was calculated using gamma band power and the potential (**g**), gamma band power only (**h**) and the potential only (**i**). Significant (*p* < 0.01) decoding accuracy is marked by non-shaded bars. Significant (*p* < 0.01) differences between mouth motor cortex-based decoding and subarea-based decoding are marked with a star

Decoding accuracy maps are shown in Fig. 5. Several electrode contacts within the mouth motor cortex had decoding accuracy significantly above chance level (corrected for multiple comparison; *q* < 0.05) in all six participants. Significant decoding accuracy was observed at all ESM-defined types of electrode contacts: tongue-, lips-, palate-, and jaw-related contacts (see Supplementary Table 1). Significant decoding accuracy was also observed in prefrontal cortex, Broca’s area and inferior parietal cortex. Overall, the best decoding accuracy was obtained using both ECoG signal features together, followed by gamma band activity only and the potential only (Fig. 6a). By using the brain signals of all electrode contacts as signal features for the multivariate decoding analysis, significant decoding accuracy (up to 85%) was observed in all participants and for each feature combination (Fig. 6b).

The results of the multivariate decoding analysis, performed by using the brain signals of all mouth motor-related electrode contacts as signal features, is shown in Fig. 6c. Significant decoding accuracy (up to approx. 80%) was observed in all participants and for each feature combination. Averaged over participants, the highest decoding accuracy was achieved using gamma band activity alone (highlighted diagonal in Fig. 6d), followed by gamma and potentials (Fig. 6e) and, with potentials alone yielding the lowest decoding accuracy (Fig. 6f).

Finally, we used the brain signals averaged over ESM-defined mouth motor cortex subareas as input signals for the decoder algorithm. The decoding accuracy of the subarea-based decoding was lower compared to the respective mouth motor cortex-based decoding for each feature and participant investigated (Fig. 6g−i). These results indicate that the signals captured with different electrode contacts within each mouth motor cortex subarea contained non-redundant information.

## Discussion

In this study, we analysed the neural signature of four distinct orofacial movement classes in the human mouth motor cortex during non-experimental, real-life behaviour with the help of ECoG. Our central finding is that each orofacial movement class, including laughing and smiling, is accompanied by a unique and sparse brain activity pattern within the ESM-defined mouth motor cortex. This indicates a previously unknown specialization of partially overlapping subareas of the motor cortex to natural mouth movement classes. Below, we discuss our ESM results, followed by our observations on movement-related brain activity and conclude with the decoding results.

Our ESM results show the typical topography of the sensorimotor cortex with a dorsal-to-ventral organization of oral effectors, i.e., the lips was represented more medial and the jaw more lateral than the tongue as previously found using various techniques, with lips represented more medial and the jaw more lateral to the tongue^3,4,33,38–42^.

Tongue and lips ESM responses were mostly described as motoric even in postcentral located electrode contacts and occupied the largest portion of the ventral sensorimotor cortex (Fig. 1a). This is in contrast to the maps of Penfield and Rasmussen^4^, but aligns well with the more recent ESM literature^38,43^. Mouth-related and even hand-related motor representations in sections of Broca’s area (area 44), as we observed here, are not yet known in the context of electrical stimulation studies, but have already been observed in several neuroimaging studies (see review^44^). We found relatively large continuous areas occupied by representations of a single oral effector, as well as spatial discontinuity, such as in the case of electrode contact B7 in P1 where distinct lips motor responses were observed separated by tongue motor responses (Fig. 2a). These observations are consistent with previous ESM research^4,43^ and indicate that spatially distant areas are supporting the same effector^19^.

Movement-related gamma modulations within mouth motor cortex were observed for all movement classes with varying strength and spatial density among participants, which may be reflecting greater volitional effort in some participants during these behaviours (see supplementary discussion).

Lip-licking events were observed surprisingly often across participants, i.e., more often than laughing and occurred mostly during communication scenarios of the participants, either with the medical personnel or with persons related to the participant. This fits very well with the literature, since lip licking is often referred to be a non-verbal communication signal, associated with nervousness^45^ or performed as a so-called flight element, i.e., being submissive to another individual^46^. It could also be performed just plainly to moisten the lips, which could happen more often in the hospital because of the dry air from the air conditioner.

However, lip-licking seems to be a common non-verbal communication signal that was observed and reported along with smiling, laughing and other non-verbal communication signals in various social scenarios, e.g., during non-verbal behaviour and deception^47^, during the learner lesson with a professional driving instructor^48^ or in the context of non-verbal cues for anxiety^49^. Motivational and affective aspects could therefore help to explain inter-individual differences, for example by decoding mood variations from intracranial EEG signals^48^.

The complexity and variability in the motor sequences of speech production taken together with the associated planning and coordination of articulation could explain the high percentage of active electrode contacts within mouth motor cortex and other associated regions during this conditions, as also previously described^49,50^. Our speech-related responses are in agreement with previous reports of gamma band activity in ECoG during experimental^5–13^ as well as non-experimental, overt speech production^12,14,51^.

Crucially, we find that each movement class was accompanied by a unique and sparse pattern of gamma band modulations within the ESM-defined mouth motor cortex. Notably, this was not reflected in the average MNI coordinates of active electrode contacts, since these were not significantly different between movement classes on the group level in any spatial direction (Fig. 1b–f). This also highlights the importance of single-subject analyses, as made possible by the high signal-to-noise ratio of intracranial EEG.

A basic pattern robustly observable across participants emerged when comparing, on the one hand, movement-related brain activity of the different classes to each other, and with the ESM-derived functional maps on the other. Comparing between classes, it became evident that each movement class was associated with a unique brain activity pattern typically. Comparing movement-related activity patterns with the ESM maps, our data show that gamma responses typically involved multiple different ESM-defined effectors mostly tongue or lips, reflecting that the same set of muscular effectors was involved across movement classes.

The amplitude of these gamma modulations differed between movement classes and also between participants (Fig. 3g) and not necessarily strongest during speech production. Thus, mouth motor cortex can indeed be involved in the control of laughing and smiling (and lip licking) during non-experimental conditions, with comparable activation strength as during natural speech production. Nevertheless, there were examples of mouth motor electrodes that recorded a decrease in gamma power (see Fig. 3d; P3 during smiling and lip licking) or participants with less pronounced gamma band effects during laughing and smiling. Beside the aforementioned emotional factors of the participants during these orofacial actions, insufficient coverage of the respective mouth motor cortex or insufficient number of events for the respective movement class might have played a role in the inter-individual differences in cortical activation/deactivation.

We characterized the pairwise spatial overlap and pairwise spatial segregation of movement class-related gamma band modulations in single participants and observed larger pairwise spatial segregation values (up to 86%) compared to the small pairwise spatial overlap values (<20%) for every possible pair of orofacial movement classes. These results point to substantial differences between the spatial brain activity patterns of the orofacial movement classes investigated here. Since 38% of the mouth motor-related electrode contacts were not active at any orofacial movement class, these results also indicate an astonishing spatial sparsity of activation of mouth motor cortex. This kind of sparse activation within the mouth motor cortex was also observed in ECoG studies in which orofacial movement-related gamma frequency maps were compared to the results of ESM, e.g., using natural speech production^14^, naming^11^ or instructed tongue movement^30,33,52^.

Our findings also show that the observed differences in movement class-related brain activity patterns are sufficiently different as to allow their correct classification on a single-trial basis. Significant decoding effects obtained from signals from single-electrode contacts were localized within the mouth motor cortex, confirming the neurophysiological origin of the decoded signals (Fig. 6a). Considerably higher decoding accuracies than from single electrodes could be achieved with multivariate decoding from all grid electrodes (Fig. 6b) and even from all mouth motor cortex channels only (Fig. 6c), demonstrating non-redundant information of different electrode contacts within this area. However, the observed non-redundancy was not trivially explainable by involvement of different effectors, as the accuracy decreased when information of averaged signals from effector-specific subareas were used (Fig. 6g-i). Together these findings suggest that high-resolution—possible micro-ECOG—recordings across the extent of mouth motor cortex might be particularly suitable to optimize the amount of decodable information about communication-related behaviour types, as proposed by Wang et al.^53^.

Summarized, we propose a movement class- or task-specific organization principle of the mouth motor cortex with sparse and action-specific recruitment of localized sub-regions. This form of action specificity in motor cortex fits well with the early idea of task specificity as a fundamental property of sensorimotor control^53–56^. Such a task-specific functional organization of the mouth motor cortex could be one reason why non-speech orofacial movement exercises may not be optimal for the treatment of speech disorders (see ref. ^57^ for a review). In contrast, language therapy methods that are based on natural behaviour-like communication scenarios with social interaction, such as Intensive Language-Action Therapy (ILAT), have been reported to significantly improve language performance even when applied late in rehabilitation programs of chronic post-stroke aphasia—possibly because they are suitable to recruit the language-specific cortical subareas as implied by our study.

The present study illustrates the feasibility and utility of investigating the neural basis of real-life human communication using intracranial EEG recordings. Besides basic insights into facets of this fundamental aspect of human behaviour, this also could help the development of neuro-technological devices designed to work based on natural brain activity during real-life situations, as proposed by Donoghue^58^. Such neuro-technological devices could translate the recorded brain signals to various outputs, which could include the following applications: simple computer displays similar to emoticons; digital avatars mimicking the intended orofacial behaviour; speech BCIs such as refs. ^59,60^ (see ref. ^27^ for a review) that may additionally use this information for the production of the corresponding intonation; or reactivating the muscle activity by electrical stimulation according to the recorded brain activity, as previously demonstrated for arm movements in monkey^61^.

## Methods

### Participants

Data sets from six participants (P1–P6) under evaluation for epilepsy surgery were included in this study. All participants were patients that had intracranial implanted electrode arrays which covered parts of the peri-Rolandic region in the left hemisphere (participant details are provided in Supplementary Table 2). The seizure onset zone was located outside of the ESM-defined mouth motor cortex in all participants, except in P4 where one electrode contact (F7) was located within the ESM-defined mouth motor cortex and lay within the seizure onset zone. This electrode contact, however, did not show any orofacial movement-related effect. All participants gave written informed consent that audio, video, and neural data obtained in the course of diagnostics could be used for scientific purposes, and the Ethics Committee of the University Medical Centre Freiburg approved the recruitment procedure.

### Data acquisition

ECoG was recorded with subdural implanted electrode arrays using a clinical AC EEG-System (IT-Med, Germany) at a sampling rate of 1024 Hz. The electrode arrays consisted of 64 platinum contacts with a diameter of 4 mm, arranged in quadratic 8 × 8 arrays at an inter-electrode contact distance of 10 mm. Recordings were hardware-filtered using a high-pass filter with a 0.032-Hz cut-off frequency and a low-pass filter at approx. 400 Hz.

Orofacial movements were identified in the digital audio and video (25-Hz frame rate) recordings, which were obtained synchronously to the ECoG. Four types of real-life orofacial movements were identified in all participants and used for further analyses: (1) lip licking, (2) speech production, (3) laughing, and (4) smiling. Note that all movements were non-instructed, although the participants were aware of being recorded for scientific purposes. The participants, who stayed at the hospital for approx. 2 weeks for the pre-neurosurgical diagnostics of epilepsy, interacted with visitors, the hospital personnel or roommates. We define this non-instructed, non-experimental behaviour during the stay at the hospital as real-life behaviour. It may not be an everyday situation and the daily life during the hospital stay certainly has special aspects, e.g., the patients have to stay in bed for the whole time (up to 2 weeks). However, due to the non-instructed, non-experimental nature of the orofacial movements, here we refer to them as real-life behaviour. We also assume that basic mechanisms of how simple orofacial movements are controlled by motor cortical regions are likely preserved even during these extraordinary hospital conditions. It should be noted that this real-life behaviour may include spontaneous as well as voluntary behaviour, i.e., we do not know if the observed orofacial movements like laughing and smiling were spontaneously or voluntarily driven.

The events used as natural orofacial movements of the type ‘speech production’ were always the beginning of a short discourse of the participants during a conversation with present dialogue partner/partners with a minimum length of 1 s and on average approx. 2.5 s. The speech production events were not further subdivided based on syntax, prosody, or other linguistic features. The topics of conversation included typical conversation topics as the weather, hospital food, soccer, politics, or television series.

We only used orofacial movement events whose beginning of the movement was clearly visible in the digital video. The very first video-frame in which a difference was recognizable compared to the frame before with regard to the respective orofacial expression was used as event onset, e.g., for lip licking the first recognizable difference was at the beginning of the slight mouth opening which occurred before the visible protrusion of the tongue. Previously, we validated the video-based approach to identify movement onset with ECoG-EMG data in a study about somatotopic mapping of natural arm and leg movement as well as speech production^14^.

We used the audio data to differentiate laughing from smiling, since these orofacial movements differ from each other due to the associated vocalization during laughing, as described in ref. ^61^. The events used as natural orofacial movements of the type ‘speech production’ were always the beginning of a short discourse of the participants during a conversation with present dialogue partner/partners with a minimum length of approx. 1 s and on average approx. 2.5 s. The speech production events were not further subdivided based on grammar, pronunciation, used articulatory organs or the like. The topics of conversation included typical conversation topics as the weather, hospital food, soccer, politics, television series or more private things. Lip-licking involved a visible protrusion of the tongue to moisten the lips, followed by a movement of the lips against each other.

Orofacial movements accompanied by clear movements of other body parts or, in the case of the non-speech events by speaking in the time period from 2 s before to 2 s after movement onset were excluded from the analysis to avoid influences of confounding movement-, or speech-related (in the case of the non-speech events) brain activity. The number of trials obtained for all participants and movement classes are summarized in Table 3.

**Table 3:** Number of trials obtained for each participant and orofacial movement class

	P1	P2	P3	P4	P5	P6	Average (mean)
Lip-licking	38	7	17	40	14	59	29
Speech production	196	148	119	89	277	44	146
Laughing	33	17	12	51	30	5	25
Smiling	57	17	42	59	59	31	44
Average (mean)	81	47	48	60	95	35	61

### Electrocortical stimulation mapping (ESM)

ESM was performed as part of the clinical diagnostics for each participant with an INOMED NS 60 stimulator (INOMED, Germany). Trains of 50-Hz pulses up to 10 s (or until the induction of stimulation effects) with alternating polarity square waves of 250-µs duration were applied to pairs of electrode contacts. Bipolar stimulation was conducted to identify non-overlapping pairs of electrode contacts with movement, sensation, and speech-related effects. Movement and sensation-related effects were normally observed after short stimulation (3–5 s), while the testing of complex speech-related effects (Token test, understanding and repeating of longer sayings) take longer and thus, the whole stimulation time of 10 s was often needed. After this, the functionally relevant contacts of the pairs were identified using the so-called monopolar ESM of individual electrode contacts against a fixed reference contact remote from regions of epileptogenic and functional relevance. Only the results of the monopolar ESM were used in the current study.

Stimulation intensity was gradually increased until the induction of a functional response, up to maximally 15 mA for mapping sensorimotor functions and up to maximally 18 mA for language mapping. Participants were unaware of the timing of stimulation until the elicitation of sensory (tactile sensations reported by the participants), motor (stimulation-evoked movement or transient inability to move), or speech-related responses (transient impairment in speech production and/or comprehension). Language areas were localized using a battery of six tasks: counting, execution of body commands, naming everyday objects, reading, repetition of sentences and a Token Test, as described by ref. ^60^.

Responses of different body parts, both sensory and motor modalities, were combined in one summary electrocortical stimulation map for each participant (e.g., for P1 in Fig. 2). In all participants the mouth motor cortex was defined based on the results of the ESM by the presence of motor responses of mouth-related body parts, namely the tongue, the lips, the palate and the jaw (see Fig. 4). In one participant (P1), lips motor responses during ESM were observed at one electrode contact close to the midline remote from all other precentral mouth motor responses. This electrode contact was assumed to cover another anatomical area, possibly the supplementary motor area, was also not active during any orofacial movement, and thus, was not used for the following analyses.

### Anatomical assignment

A T1-weighted magnetization-prepared rapid-acquisition gradient-echo (MPRAGE) data set with 1-mm isotropic resolution was obtained during the implantation period from each participant on a 1.5-T Vision magnetic resonance imaging (MRI) scanner (Siemens, Erlangen, Germany). This MRI data set was normalized to MNI space using SMP8^62^. The implanted electrode arrays, as well as the individual positions of the central and lateral sulcus were identified and marked manually. Then, a hierarchical anatomical assignment of electrode contacts to cortical areas of each participant was performed using the topographic information about the individual position and extent of the central and lateral sulci (cf. refs. ^14,63^). The MNI-based anatomical probability information was then obtained for contacts coordinates for each electrode using the SPM Anatomy Toolbox V18^64^.

### Data pre-processing

For data pre-processing, the recorded ECoG was re-referenced to a common average reference (CAR) over all implanted electrode contacts of the respective electrode array, as used in ECoG studies to remove common noise^29,65–69^. Data epochs were generated from −2 s to +2 s around the onset of each event. In the following, these data epochs are referred to as trials.

For computation of the ERPs, each trial was baseline-corrected by subtracting the median potential across trials and across the time period from −1.5 s to −1 s with respect to event onset. The average potential change of each electrode contact was then determined as the median across the baseline-corrected trials.

For the spectral analysis, a multi-taper method^70,71^ with three Slepian tapers, a time window of 200 ms length and 20-ms time steps was used. As for the potentials, the baseline period was defined as from −1.5 s to −1 s relative to the onset of the respective event. Relative changes in spectral power were calculated by dividing the time-resolved amplitudes of each trial and frequency bin by the median baseline power of the respective frequency. Average relative spectral power was then calculated by taking the median over trials for each time-frequency bin.

### Topographic maps

For the topographical analysis of event-related gamma band activity, the average data in the frequency range of 55–200 Hz and in a time window of 0–500 ms after event onset were used, as earlier ECoG studies observed robust movement- and speech production-related gamma band modulations in this frequency range and at this latency^7,12,14,51,66,72–75^. To test for significance, a sign test was used, corrected for multiple comparisons across the number of electrode contacts (64 contacts per participant). The significance threshold was chosen depending on the number of events per movement class of the respective participant (Table 3), and was *p* < 0.05 < 100 events and *p* < 0.0001 for >100 events. To correct for multiple comparisons, we used the false discovery rate (FDR) approach by ref. ^76^ or the Bonferroni correction in case the FDR correction resulted in a threshold that was stricter than the one corresponding to the Bonferroni-corrected test. ERP changes were analysed in the same time window (0–500 ms) and with the same statistical tests.

### Spatial distribution of event-related gamma modulations

We investigated the spatial distribution of the gamma power modulations of the four movement classes on the group level in MNI space, including all electrode contacts that were both located within the ESM-defined mouth motor cortex and showed significant (*p* < 0.01) gamma power modulations in at least one movement class. We refer to electrode contacts that recorded significant brain activity as active electrode contacts. In this specific analysis, we did not correct for multiple comparisons like we did in the other analyses, since the number of electrode contacts was already reduced to contacts located within the mouth motor cortex. The locations of active electrode contacts were visualized on a standard brain surface (see Fig. 2a). Differences in average position between active electrode contacts of different movement classes were statistically evaluated using the MNI coordinates of the respective electrode contacts. We tested for significant difference in each spatial direction in MNI space using a two-sided Wilcoxon rank sum test at a significance threshold of *p* < 0.01.

### Overlap/segregation analysis on the single-electrode level

One of the main questions addressed in the present study was to what degree brain activity at individual electrode contacts was similar or different when comparing the four different movement classes. To address this question, we performed an overlap/segregation analysis with the following steps: First, based on their statistical significance, gamma band modulations of each event and for every electrode contact within the mouth motor cortex were classified in three distinct categories: (I) significant (*p* < 0.01; uncorrected), (II) intermediate (not significant, but *p* < 0.3), and (III) insignificant (*p* > 0.3).

For each participant, the *overlap* of a pair of movement classes was defined as the percentage of all electrode contacts in the mouth motor cortex that both fell in class I or both fell in class II. Thus, an electrode contact that was insignificant in both conditions would not be counted as an overlap. *Segregation* of two different movement classes was defined as the percentage of electrode contacts located within the mouth motor cortex that fell into different classes, including class III. Thus, for example an electrode contact that recorded significant gamma band modulation in one condition and insignificant modulation in the other would be counted as segregation. We additionally calculated segregation and overlap using only pairs of movement classes with more than 30 trials to exclude effects that could have resulted from small sample sizes.

### Single-trial decoding analysis

We used a multivariate decoder algorithm to investigate the single-trial discriminability of the four natural movement classes. To this aim, we used a regularized linear discriminant analysis (rLDA)^63^ with leave-one-out cross-validation and evaluated the decodable information in different ECoG signal features or a combination of signal features, respectively: (i) gamma band power (average over the time-frequency window from 55–200 Hz and 0–500 ms) per trial and electrode contact, (ii) movement-related potential (average over the time window from 0–500 ms) per trial and electrode contact and (iii) a feature combination of both (i) and (ii), i.e., using two feature values per trial and electrode contact.

Since different sample sizes existed for each orofacial movement class, we first of all calculated the decoding accuracy for each class separately and then averaged the decoding accuracy over the four classes. A permutation test was used to determine the statistical significance of the decoding accuracy. To this end, we generated 10,000 sequences containing the class labels with the same proportions as the actual data but in a randomized order. For each sequence, a random decoding accuracy was calculated as for the actual data. Then, the *p* value was calculated as the ratio of the random decoding accuracy values that were equal to or higher than the real decoding accuracy. The false discovery rate (FDR) procedure by ref. ^76^ was used to correct for multiple tests.

In addition to this single-electrode decoding analysis, we also performed decoding analyses, in which all features across the whole grid (Fig. 6b) or the ESM-defined mouth motor cortex (Fig. 6c) were concatenated and the rLDA was trained on these feature vectors. The aim of this analysis was to test whether different electrode contacts contain non-redundant information, possibly yielding an increased decoding accuracy. These analyses were also performed with different ECoG signal features (i and ii) or the combination of both (iii), as was done for single-electrode contacts. Note that for this kind of multivariate decoding analysis, the preselection of significant channels from the single-electrode-based decoding would bear the risk of overfitting and exaggerated results^77^. The confusion matrices of the mouth motor cortex-based decoding were calculated as the mean over participants for each signal feature. For each orofacial movement class, the respective row indicates the percentage of classifications for the movement class in the respective column (Fig. 6d−f).

In a final decoding analysis, we averaged the signal features over electrodes within mouth motor cortex subareas, which were defined based on the specific ESM mouth-related motor responses (the tongue, the lips, the palate or the jaw). The reason of this final decoding analysis was that, if each subarea was activated uniformly across the four different orofacial movement classes, decoding from the averaged subarea-data would increase the decoding accuracy due to the out-averaging of noise. In contrast, if each subarea was activated differently across the four orofacial movement classes, decoding from the averaged subarea-data would rather decrease the decoding accuracy due to the out-averaging of the class-discriminative signal.

### Reporting Summary

Further information on experimental design is available in the Nature Research Reporting Summary linked to this article.

## Supplementary information

Reporting Summary

Supplementary Information

## Data Availability

Any code used in this study is available from the authors upon request.

## Data availability

The data that support the findings of this study are available from the corresponding authors upon request.
