# Author Correction: Human motor cortex relies on sparse and action-specific activation during laughing, smiling and speech production

**DOI:** 10.1038/s42003-019-0404-8

**Published:** 2019-04-08

**Authors:** Markus Kern, Sina Bert, Olga Glanz, Andreas Schulze-Bonhage, Tonio Ball

**Affiliations:** 1 Medical AI Lab, Department of Neurosurgery, Medical Center – University of Freiburg, Freiburg, 79106 Germany; 2 Neurobiology and Biophysics, Faculty of Biology, University of Freiburg, Freiburg, 79104 Germany; 3 Epilepsy Center, Department of Neurosurgery, Medical Center – University of Freiburg, Freiburg, 79106 Germany; 4 BrainLinks-BrainTools Cluster of Excellence, University of Freiburg, Freiburg, 79110 Germany; 5 Hermann Paul School Linguistics, University of Freiburg, Freiburg, 79085 Germany; 6 GRK 1624, University of Freiburg, Freiburg, 79098 Germany

**Keywords:** Motor cortex, Brain-machine interface

Correction to: *Communications Biology* 10.1038/s42003-019-0360-3, Published online 26 March 2019

In the original version of the paper, Fig. 4 contained an error in the labels for “Intermediate” and “Insignificant” in the figure key, making the symbols partially illegible. This error has been corrected in the HTML and PDF versions of the paper.

