# Supplementary material for: Human motor cortex relies on sparse and action-specific activation during laughing, smiling and speech production
Source: Commun Biol. 2019 Mar 26;2:118. doi: 10.1038/s42003-019-0360-3 (PMC6435746; doi:10.1038/s42003-019-0360-3)
Supplement: Supplementary file 1 — Reporting Summary [file 42003_2019_360_MOESM1_ESM.pdf]

nature research

Corresponding author(s): Tonio Ball

Reporting Summary

Nature Research wishes to improve the reproducibility of the work that we publish. This form provides structure for consistency and transparency in reporting. For further information on Nature Research policies, see Authors & Referees and the Editorial Policy Checklist.

Statistical parameters

When statistical analyses are reported, confirm that the following items are present in the relevant location (e.g. figure legend, table legend, main text, or Methods section).

n/a	Confirmed	
☐	☒	The exact sample size (n) for each experimental group/condition, given as a discrete number and unit of measurement
☐	☒	An indication of whether measurements were taken from distinct samples or whether the same sample was measured repeatedly
☐	☒	The statistical test(s) used AND whether they are one- or two-sided<br>*Only common tests should be described solely by name; describe more complex techniques in the Methods section.*
☐	☒	A description of all covariates tested
☐	☒	A description of any assumptions or corrections, such as tests of normality and adjustment for multiple comparisons
☐	☒	A full description of the statistics including central tendency (e.g. means) or other basic estimates (e.g. regression coefficient) AND variation (e.g. standard deviation) or associated estimates of uncertainty (e.g. confidence intervals)
☐	☒	For null hypothesis testing, the test statistic (e.g. F, t, r) with confidence intervals, effect sizes, degrees of freedom and P value noted<br>*Give P values as exact values whenever suitable.*
☒	☐	For Bayesian analysis, information on the choice of priors and Markov chain Monte Carlo settings
☐	☒	For hierarchical and complex designs, identification of the appropriate level for tests and full reporting of outcomes
☐	☒	Estimates of effect sizes (e.g. Cohen's d, Pearson's r), indicating how they were calculated
☐	☒	Clearly defined error bars<br>*State explicitly what error bars represent (e.g. SD, SE, CI)*

Our web collection on statistics for biologists may be useful.

Software and code

Policy information about availability of computer code

Data collection	We used the "Coherence Software" from Neurolite to detect orofacial movements in the digital audio and video recordings and to mark the timepoints of the respective event onset in the synchronously recorded ECoG.
Data analysis	For data pre-processing and data analysis we used Matlab (version 2017a) and the anatomy toolbox version 18 of SPM8. For the hierarchical anatomical assignment of electrode contacts to cortical brain areas we used a custom Matlab-based algorithm. Custom code is available from the corresponding author upon request.

For manuscripts utilizing custom algorithms or software that are central to the research but not yet described in published literature, software must be made available to editors/reviewers upon request. We strongly encourage code deposition in a community repository (e.g. GitHub). See the Nature Research guidelines for submitting code & software for further information.

April 2018

Data

Policy information about availability of data

All manuscripts must include a data availability statement. This statement should provide the following information, where applicable:

- Accession codes, unique identifiers, or web links for publicly available datasets
- A list of figures that have associated raw data
- A description of any restrictions on data availability

No unique materials were used.

Field-specific reporting

Please select the best fit for your research. If you are not sure, read the appropriate sections before making your selection.

☒ Life sciences ☐ Behavioural & social sciences ☐ Ecological, evolutionary & environmental sciences

For a reference copy of the document with all sections, see nature.com/authors/policies/ReportingSummary-flat.pdf

Life sciences study design

All studies must disclose on these points even when the disclosure is negative.

Sample size	Since we used data from non-experimental behavior, the sample size of each orofacial movement class was determined by the amount of suitable events from the respective participant. Participants were patients undergoing evaluation for epilepsy surgery and thus, we could only use data from patients whith grid electrode contacts over the mouth motor cortex in the time window of evalution. We think that the sample sizes are sufficient for the kind of analysis we did, especially since we adapted our methods to address the issue of possible small sample sizes.
Data exclusions	We excluded orofacial movements accompanied by clear movements of other body parts or, in the case of the nonspeech events by speaking in the time period from two seconds before to two seconds after movement onset from the analysis to avoid influences of confounding movement-, or speech-related brain activity.<br>We also excluded one electrode contact in P1 from the further analysis. At this electrode contact lips motor responses were observed during ESM, but this contact was located close to the midline remote from all other precentral mouth motor responses. At this contact, no significant brain activity was recorded during any orofacial movement class investigated. We assume that this contact covers another anatomical area, possibly the supplementary motor area, and thus, we dropped it from the following analyses.
Replication	Our basic findings were reproducible across all six investigated participants.
Randomization	Since we used orofacial movements-related ECoG data during non-experimental, real-life conditions, no randomization was used. Orofacial movements within each investigated participant were arranged chronologically in their order of appearance in the ongoing ECoG recordings.
Blinding	Since we did not allocate patients to different groups, no blinding was neccessary.

Reporting for specific materials, systems and methods

Materials & experimental systems

n/a	Involved in the study	
☒	☐	Unique biological materials
☒	☐	Antibodies
☒	☐	Eukaryotic cell lines
☒	☐	Palaeontology
☒	☐	Animals and other organisms
☐	☒	Human research participants

Methods

n/a	Involved in the study	
☒	☐	ChIP-seq
☒	☐	Flow cytometry
☒	☐	MRI-based neuroimaging

Human research participants

Policy information about studies involving human research participants

Population characteristics	We analyzed ECoG data of 6 participants (4 male, 2 female), aged between 22 and 57, who were diagnosed with pharmaco-resistent epilepsy and thus, were undergoing pre-neurosurgical evaluation with ECoG. The ESM-defined mouth motor cortex on

the left hemisphere was at least partially covered in all participants investigated. The seizure onset zone was located outside of the investigated mouth motor cortex in all participants, except in P4 where one electrode contact was located within the seizure onset zone, but did not show any movement-related effect.

Recruitment	We collected all data retrospectively with the help of digital audio and video data after the neurosurgical procedures were completed. Thus, we have chosen participants whose mouth motor cortex was at least partially covered by an electrode grid due to the pre-surgical diagnosis. We have no inforamtion about the genotype or the current diagnosis of these participants. Participant details are provided in Supplementary Table 1.
