# Supplementary material for: Human motor cortex relies on sparse and action-specific activation during laughing, smiling and speech production
Source: Commun Biol. 2019 Mar 26;2:118. doi: 10.1038/s42003-019-0360-3 (PMC6435746; doi:10.1038/s42003-019-0360-3)
Supplement: Supplementary file 2 — Supplementary Information [file 42003_2019_360_MOESM2_ESM.pdf]

Supplementary Table 1: Results from decoding movement classes from single mouth motor-related electrode contacts, using gamma band activity (GA), potential (Pot) and both together (Pot+GA) as signal features. *Four different mouth motor function-related electrode types (tongue, lips, palate and jaw) were defined based on the results of the ESM. For each patient, electrode type and used signal feature, the number of electrodes with significant DA, the average DA, and the maximal DA are shown.*

		**Tongue**			**Lips**			**Palate**			**Jaw**		
		GA	Pot	Pot + GA	GA	Pot	Pot + GA	GA	Pot	Pot + GA	GA	Pot	Pot + GA
Sig. Elec.	P1	5/7	5/7	5/7	2/4	2/4	3/4	1/1	1/1	1/1	-	-	-
	P2	0/1	0/1	0/1	0/1	0/1	1/1	-	-	-	1/1	0/1	1/1
	P3	3/6	3/6	4/6	1/1	0/1	0/1	-	-	-	-	-	-
	P4	4/6	0/6	4/6	3/8	1/8	5/8	-	-	-	-	-	-
	P5	5/8	4/8	8/8	-	-	-	0/1	0/1	1/1	-	-	-
	P6	1/5	0/5	1/5	4/10	3/10	3/10	-	-	-	-	-	-
Av. DA	P1	40%	34%	41%	34%	32%	33%	43%	43%	47%	-	-	-
	P2	23%	18%	31%	35%	27%	35%	-	-	-	37%	31%	37%
	P3	35%	34%	36%	41%	29%	27%	-	-	-	-	-	-
	P4	33%	28%	37%	32%	31%	35%	-	-	-	-	-	-
	P5	35%	33%	39%	-	-	-	25%	31%	32%	-	-	-
	P6	31%	27%	35%	35%	33%	37%	-	-	-	-	-	-
Max. DA	P1	48%	43%	52%	40%	40%	43%	43%	43%	47%	-	-	-
	P2	23%	18%	31%	35%	27%	35%	-	-	-	37%	31%	37%
	P3	45%	40%	41%	41%	29%	27%	-	-	-	-	-	-
	P4	42%	32%	45%	39%	35%	45%	-	-	-	-	-	-
	P5	45%	41%	48%	-	-	-	25%	31%	32%	-	-	-
	P6	46%	35%	50%	49%	51%	59%	-	-	-	-	-	-

Supplementary Table 2: Overview of the patients (P1–P6) included in this study.

	**age**	**sex**	**handed-ness**	**grid location**	**lesion**	**seizure onset**
**P1**	41	F	left	64-contact grid left fronto-temporo-parietal	focal cortical dysplasia left fronto-central cortex	left precentral
**P2**	57	M	ambi-dexterity	64-contact grid left fronto-temporo-parietal	hippocampal sclerosis left; lesion left temporal cortex, left frontal cortex	left parieto-occipital
**P3**	27	M	right	64-contact grid left fronto- parietal	focal cortical dysplasia, type Ib (Palmini), left frontal cortex	left frontal
**P4**	27	M	right	64-contact grid left fronto-temporo-parietal	focal cortical dysplasia, type Ib (Palmini), left frontal cortex	left frontal
**P5**	55	M	right	64-contact grid left fronto-temporo-parietal	lesion left frontal	left frontal
**P6**	49	F	right	64-contact grid left fronto-temporo-parietal	focal complex cortical dysplasia, left fronto-central cortex	left frontal

Supplementary discussion

Even though such volitional effort would be difficult to quantify retrospectively, it may be a noteworthy observation that P1 often answered the question of her boyfriend: “How are you? How do you feel?” with a smile or laughing and a following “Everything is ok”. However, without her boyfriend nearby, it became clear that she was having a tough time at the hospital, and that she was not experiencing her situation as genuinely “ok”. The laughing and smiling data of P1 could solely be obtained when her boyfriend was talking to her, and it appeared to us that her smiling and laughing might have been feigned to soothe him down. One could speculate that this fits well with the pronounced gamma band effects in the orofacial motor cortex of this patient. In contrast, P5 seemed to be good-tempered and only slightly emotionally affected by the whole presurgical diagnostic procedure. This became noticeable because he was often joking with the medical personnel. It appeared that his smiling and laughing was more emotionally-driven, produced without much volitional effort and actually reflecting his natural buoyancy. Compared to the gamma band effects in the mouth motor cortex of P1, the smiling- and laughing-related gamma band effects of P5 were considerably less pronounced. These two examples let us speculate that emotional factors might indeed have played a role in the striking inter-individual differences in cortical activation during real-life smiling and laughing in our data.
